# Tracking and Behavior Analysis of Group-Housed Pigs Based on a Multi-Object Tracking Approach

**DOI:** 10.3390/ani14192828

**Published:** 2024-09-30

**Authors:** Shuqin Tu, Jiaying Du, Yun Liang, Yuefei Cao, Weidian Chen, Deqin Xiao, Qiong Huang

**Affiliations:** 1 College of Mathematics and Informatics, South China Agricultural University, Guangzhou 510642, China; tsq5_6@scau.edu.cn (S.T.); dujiaying2001@163.com (J.D.); cyf_1208@163.com (Y.C.); chenwd2543@163.com (W.C.); deqinx@scau.edu.cn (D.X.); 2 Guangzhou Key Laboratory of Intelligent Agriculture, Guangzhou 510000, China

**Keywords:** pig behavior tracking, OC-SORT, group-housed pigs, YOLOv8, behavior analysis

## Abstract

**Simple Summary:**

This study developed a new method for automatically tracking and analyzing pig behavior in complex environments. We use the YOLOv8 algorithm for real-time detection and behavior classification, and employ the OC-SORT algorithm to address tracking issues caused by lighting changes, occlusions, and collisions between pigs. This method enables the automatic analysis of behavior, helping farm managers promptly detect abnormalities and health issues in pigs. Experimental results show that the method performs excellently in behavior recognition and tracking, accurately recording pig behavior to provide technical support for monitoring the health and welfare of pig herds.

**Abstract:**

Smart farming technologies to track and analyze pig behaviors in natural environments are critical for monitoring the health status and welfare of pigs. This study aimed to develop a robust multi-object tracking (MOT) approach named YOLOv8 + OC-SORT(V8-Sort) for the automatic monitoring of the different behaviors of group-housed pigs. We addressed common challenges such as variable lighting, occlusion, and clustering between pigs, which often lead to significant errors in long-term behavioral monitoring. Our approach offers a reliable solution for real-time behavior tracking, contributing to improved health and welfare management in smart farming systems. First, the YOLOv8 is employed for the real-time detection and behavior classification of pigs under variable light and occlusion scenes. Second, the OC-SORT is utilized to track each pig to reduce the impact of pigs clustering together and occlusion on tracking. And, when a target is lost during tracking, the OC-SORT can recover the lost trajectory and re-track the target. Finally, to implement the automatic long-time monitoring of behaviors for each pig, we created an automatic behavior analysis algorithm that integrates the behavioral information from detection and the tracking results from OC-SORT. On the one-minute video datasets for pig tracking, the proposed MOT method outperforms JDE, Trackformer, and TransTrack, achieving the highest HOTA, MOTA, and IDF1 scores of 82.0%, 96.3%, and 96.8%, respectively. And, it achieved scores of 69.0% for HOTA, 99.7% for MOTA, and 75.1% for IDF1 on sixty-minute video datasets. In terms of pig behavior analysis, the proposed automatic behavior analysis algorithm can record the duration of four types of behaviors for each pig in each pen based on behavior classification and ID information to represent the pigs’ health status and welfare. These results demonstrate that the proposed method exhibits excellent performance in behavior recognition and tracking, providing technical support for prompt anomaly detection and health status monitoring for pig farming managers.

## 1. Introduction

With the development of intelligent and efficient animal farming, the automatic monitoring of pig health plays a crucial role in the modern livestock industry. Precision livestock farming technology uses advanced sensors and data analytic technology to monitor individual animals in real-time, which provides great potential to improve management efficiency and production. The specific activity patterns of farmed animals, including standing, lying, and eating, evidently reflect the consequences of compromised health and welfare. Due to the large animal-to-staff ratio, conducting manual observation or recording animal behavior is labor-intensive, subjective, and inefficient. Additionally, human presence can change animal behavior, making monitoring without humans in the barn necessary for accurate observations. Therefore, there is an urgent need for effective automatic monitoring methods to accurately track and analyze pig behaviors under different environmental conditions. This not only helps in the timely detection of abnormalities such as diseases, stress, or environmental factors, but also enhances the efficiency of pig farming [1,2].

Regarding existing automated animal monitoring systems, there are two categories. Firstly, there are contact-based methods with attached sensors, such as utilizing a high-frequency radio frequency identification (HF RFID) system to register drinking behavior [3], employing accelerometers for the detection of abnormal gait patterns [4], using pressure pads to identify limping behavior [5], and using accelerometry and GNSS data to classify animal behavior [6]. Considering the cost of hardware and maintenance on large-scale commercial farms, contact-based solutions for automated tracking are not preferable. Secondly, there is contactless monitoring using computer vision and deep learning technologies [7,8,9], which has enjoyed growing popularity due to its low cost and sustainability compared to contact-based sensors. For example, Duc Duong Tran et al. proposed a method using deep learning for automatically monitoring and detecting abnormalities in pig behaviors [10]. Zhang et al. introduced a transformer-based neural network (TNN) model for recognizing pig feeding behavior and detecting potential dangers [11]. Alameer et al. utilized deep learning technology to identify pig postures and drinking behaviors and used drinking behavior as an indicator of pig health status [12]. Computer vision technology enables the real-time monitoring and identification of pig behavior through image processing, achieving accurate health monitoring. Further research is needed to extract behavioral information from surveillance videos in pig farms for improved monitoring [13].

In video surveillance, tracking and behavior recognition are crucial components for group-housed pig monitoring applications. In the field of pig tracking research, various methods have been proposed to achieve an accurate tracking performance. For instance, Chen et al. introduced a real-time detection and tracking method based on YOLACT, successfully detecting and tracking the various major body parts of pigs, thereby providing robust support for pig behavior analysis [14]. Aggaluck et al. achieved accurate pig tracking by training a faster region-based convolutional neural network to identify the body and head of pigs [15]. Gong et al. proposed an improved IOU-Tracker pig tracking algorithm, incorporating the YOLOv5s network for the real-time detection and tracking of pigs [16]. Zhang et al. employed a tracking method based on discriminative correlation filters for the rapid tracking of multiple pigs [17]. Tu et al. proposed an enhanced DeepSORT target-tracking algorithm that integrates the YOLOX-S and YOLOv5s detectors. By focusing on trajectory processing and data association tailored to pig-specific scenarios, they achieved significant improvements in tracking stability [18]. In the realm of pig behavior recognition, researchers have also made significant strides. Zhou et al. introduced a method for individual pig identification using the three-dimensional (3D) point clouds of the pig’s back surface, successfully addressing the challenge of difficult sample collection in pig face recognition [19]. Hao et al. proposed a novel deep mutual learning enhanced two-stream pig behavior recognition approach. Their model integrates two mutual learning networks utilizing RGB and optical flow streams, with each branch comprising collaboratively learning student networks to capture a robust appearance and motion features. This innovative method effectively improves the performance of pig behavior recognition [20]. To investigate aggressive behaviors in group-housed pigs, Gao et al. presented a hybrid model that combines convolutional neural network (CNN) and gated recurrent unit (GRU) to distinguish between aggressive behaviors and other behaviors in monitored videos [21]. Ji et al. proposed a method that employs the temporal shift module (TSM) for the automatic recognition of pig aggression. This approach enhances the accuracy of identifying aggressive behaviors in pigs by allowing the model to effectively process both spatial and temporal features [22]. These research methods have allowed significant progress in pig health monitoring. However, there are still considerable limitations when dealing with complex behavior analysis in pigs, especially in specific scenarios. For instance, collisions between pigs can easily result in target loss, making it challenging to effectively track pig targets for long periods. In commercial automated farming settings, variations in lighting conditions and dense occlusion can lead to frequent changes in target IDs during the tracking process.

Existing research primarily focuses on the identification or automatic tracking of pig behaviors, lacking specific behaviors’ statistics and analysis. And, the Kalman filter (KF) of the current mainstream MOT methods is susceptible to noise when utilizing linear motion for target position estimation, resulting in the inaccurate estimation of the motion direction. The deviations in KF parameters also result in frequent target loss due to occlusions. Additionally, there are few studies discussing the work of recovering the lost trajectory for re-tracking, which has played an important role in long-time tracking.

To address these issues, we propose a pig tracking and behavior recognition algorithm based on V8-Sort to analyze the different behavioral statuses of group-housed pigs. Firstly, the algorithm employs the YOLOv8 detection algorithm for pig detection and behavior classification. Secondly, the OC-SORT algorithm is utilized for pig tracking under challenging scenarios. To reduce the impact of noise and occlusion during pig tracking tasks, OC-SORT employs observation-centered momentum (OCM), re-observation update (ORU), and recovery (OCR) strategies. Especially, when a target is lost for re-tracking, OC-SORT can employ the ORU strategy for target adjustment through virtual trajectories and utilize a second correlation to recover the lost trajectory. Finally, we create an automated behavior analysis algorithm, which records the duration of each behavior for each pig in each pen. To validate the effectiveness of V8-Sort, we compare it with current mainstream tracking algorithms, including Trackformer [23], JDE [24], and TransTrack [25], on the same dataset. Furthermore, a comprehensive evaluation of the proposed tracking algorithm is conducted on four ten-minute video datasets.

The main contributions of this work are as follows:

(1) A pig behavior tracking algorithm based on V8-Sort is proposed to decrease noise and improve the robustness to occlusions and nonlinear motions.

(2) The V8-Sort is validated on four ten-minute and one sixty-minute video datasets, exploring the work of recovering the lost trajectory for re-tracking.

(3) An automatic behavior analysis algorithm is designed to record the duration of four types of behaviors for each pig in each pen based on behavior information from tracking results.

(4) The effectiveness of the OC-SORT tracker is demonstrated through comparative experiments compared with other mainstream trackers.

## 2. Materials

The study utilizes two distinct datasets. The first dataset, provided by Posta et al. [26], is a publicly available dataset comprising 19 one-minute video clips and 4 ten-minute video clips. The videos cover a variety of complex environments, including different pig quantities, different pig ages, and lighting conditions. For training, 6 one-minute video clips are randomly selected, while the remaining clips are used for testing. The second dataset is a private dataset captured from a commercial pig farming facility in Foshan City. It consists of 19 one-minute video clips and 1 one-hour video clip, with 10 used for training and 10 used for testing. The resolution of these videos is 2688 × 1520, and each video is captured and annotated at a rate of 5 frames per second (fps).

Both datasets encompass scenes occurring during day and night, in crowded and sparse conditions, as well as in scenarios with varying levels of pig activity, ranging from frequent(H) to moderate(M) and less(L) active situations. Simultaneously, based on manual observation, videos with a higher quantity of pigs and significant occlusion due to the pigs clustering together are categorized as dense videos, whereas videos having less occlusion are categorized as sparse videos. The pigs are of different ages, categorized into a nursery period (3–10 weeks), growing period (11–18 weeks), and finishing period (19–26 weeks). Additionally, according to growth curves, the weight varies across different age stages. Pigs in the nursery period (3–10 weeks) typically weigh between 7 and 25 kg. During the growing period (11–18 weeks), they weigh around 25–70 kg. And in the finishing period (19–26 weeks), their weight usually ranges from 70–to 130 kg. The specific description of the test dataset is outlined in Table 1.

**Figure 1:**
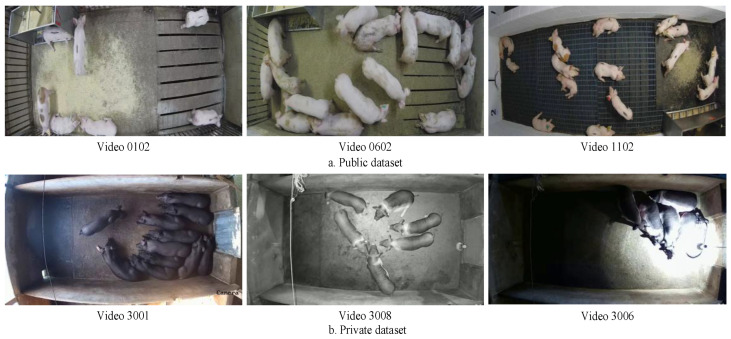
Part of group-housed pig images.

Some example images of group-housed pigs are illustrated in Figure 1. To ensure the diversity of pig behavior data, we extracted key frames using the Ffmpeg6.0 [27] software, and used the Darklabel [28] software to annotate pig behavior classifications, including standing, lying, eating, and other behaviors. The pigs are categorized into two breeds: white pigs and black-spotted pigs. Each pig is allocated approximately 1.2 square meters of pen space, and temperature and humidity within the barn are controlled to ensure suitable environmental conditions for the animals. Access to both public and private datasets facilitates a comprehensive analysis and validation of pig behavior across different environments. By analyzing various pig behaviors, we can gain deeper insights into their behavioral status in different scenarios, which is crucial for improving livestock management and enhancing production efficiency.

## 3. Methods

**Figure 2:**
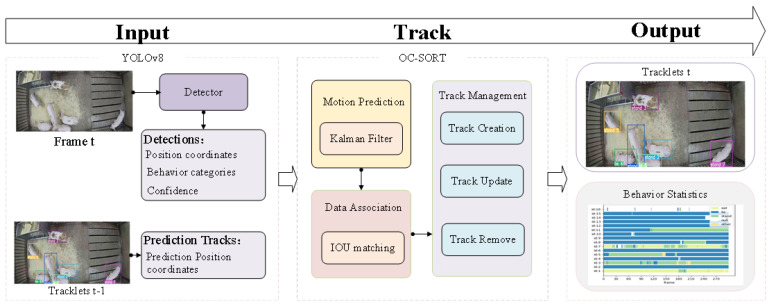
The overall structure of V8-Sort.

This study introduces the V8-Sort for the target tracking and behavior analysis of group-housed pigs. The overall structure is illustrated in Figure 2. First, YOLOv8 is employed as the detector to complete four tasks including target detection, displaying the target’s bounding box positions, behavior categories, and confidence scores. Secondly, the OC-SORT algorithm is utilized to track each pig to reduce the impact of pigs’ adhesion and occlusion on tracking. It contains three key tasks: motion prediction, data association, and track management. Finally, we create the behavior analysis method to calculate the frequency of each behavior for each pig based on the pig’s ID and categories information. Therefore, the behavioral statistics information for all pigs is yield.

### 3.1. Pig Detection Based on the YOLOv8n Model

**Figure 3:**
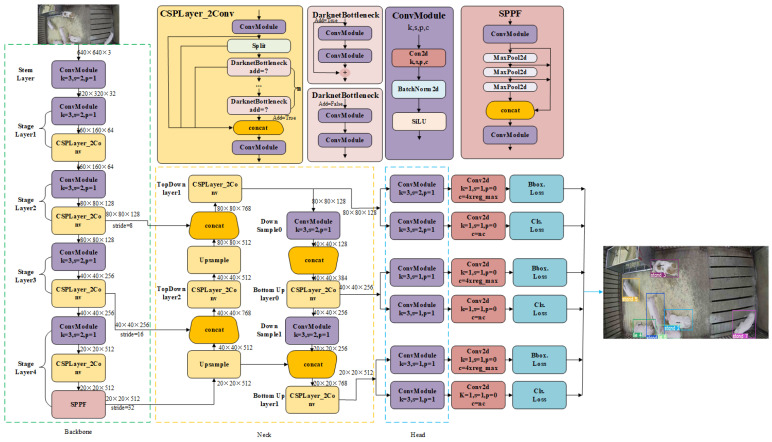
The pipeline of the YOLOv8n algorithm.

The YOLOv8n algorithm is employed as the target detector for object detection, and its model structure is primarily divided into three components: (i) Backbone for extracting image features; (ii) Neck for fusing multi-scale feature information; (iii) Prediction for predicting confidence, category, and anchor boxes. The pipeline of the YOLOv8n is shown in Figure 3. The backbone of YOLOv8n consists of 10 layers and is organized into 5 sections, namely Stem layer, Stage layer1, Stage layer2, Stage layer3, and Stage layer4. The Neck part consists of 7 layers, which are divided into 6 parts, including TopDown layer1, TopDown layer2, Down Sample0, Bottom Up layer0, Down Sample1, and Bottom Up layer1. The Head part is composed of 6 layers and divided into 3 parts, each of which consists of ConvModule, Con2d, Bbox.Loss, and Cls.Loss. The functional descriptions of each layer can be found in Table 2. YOLOv8n is trained with specific parameters, including 80 epochs, an intersection over union (IOU) threshold of 0.7, and a default confidence threshold of 0.25. Compared to older versions of the YOLO algorithm, YOLOv8n maintains high speed and accuracy while also offering greater versatility, flexibility, and simplicity.

### 3.2. Pig Tracking Based on the OC-SORT Algorithm

**Figure 4:**
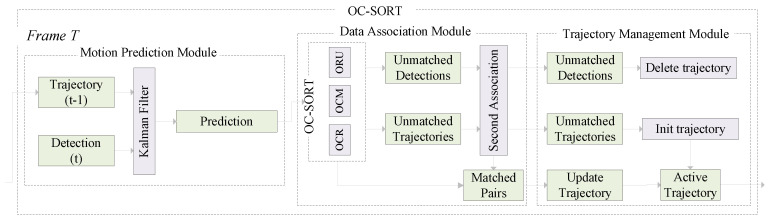
The flowchart of OC-SORT.

To reduce the impact of noise, including visual disturbances such as lighting changes and shadows, environmental interferences from surrounding movements, and data inaccuracies during the pig tracking task, we employ the OC-SORT to complete the pig’s real-time tracking and behavior analysis tasks in video streams. Figure 4 shows the flowchart of the OC-SORT algorithm. The algorithm consists of three key modules: motion prediction, data association, and trajectory management. First, OC-SORT uses the KF to achieve the dynamic prediction of target trajectories in the motion prediction module. Then, it employs a data association algorithm to associate the detection targets and tracks across different frames of data. Finally, the trajectory management module of OC-SORT performs the real-time updating of trajectory information and status maintenance of the associated targets to ensure the accurate and coherent tracking and behavioral analysis of the pigs. The specific tracking process is as follows.

#### 3.2.1. Pig Motion Prediction

The core KF within the motion prediction module is utilized to achieve the state prediction of pig trajectories. First, we define a seven-dimensional state vector x, as shown Equation (1) to represent the trajectory state of the pigs.

(1) $$x=\left(u,v,s,r,\hat{u},\hat{v},\hat{s}\right)$$

where $\left(u,v\right)$ represents the center position coordinates of the target, s is the bounding box scale (area), and r is the bounding box aspect ratio. The aspect ratio r is assumed to be a constant value. The other three variables, $\hat{u},\hat{v}$, and $\hat{s}$ are the corresponding time derivatives.

Then, the previous frame’s state estimation and the current frame’s detection results are input to the KF prediction module to obtain an accurate estimation of the target’s state in the current frame. And, it is obtained using the state estimation ($x_{t|t-1}$) and the covariance matrix ($P_{t|t-1}$) by Equations (2) and (3). Equations (2) and (3) are shown as follows:

(2) $$x_{t|t-1}=F_{t}x_{t-1|t-1}$$

(3) $$P_{t|t-1}=F_{t}P_{t-1|t-1}F_{t}^{T}+Q_{t}$$

where $x_{t|t-1}$ is the posterior state estimate at the current frame, $x_{t-1|t-1}$ is the posterior state estimate in the previous frame. P is the posterior estimate covariance matrix, F is the state transition model, and Q is the process noise. After predicting the mean x and covariance P of the target in the next frame using KF, the matching operation is performed between trajectories with the newly detected targets in the next frame.

#### 3.2.2. Data Association

Data association enables the continuity of association for the same pig in continuous frames, ensuring consistency in pig ID value. The Hungarian algorithm [29] is employed for data association matching between detected pigs and tracks. We utilize the OCM strategy of OC-SORT to incorporate the direction consistency of trajectories in the associated cost matrix operation, facilitating improved matching between tracking trajectories and observation results. After the usual association stage, the OCR technique of OC-SORT will initiate a second attempt to associate the final observation results of unmatched tracks with the unmatched observation values. Given N existing tracks and M detections, the association cost matrix is formulated as in Equation (4).

(4) $$C\left(\hat{X},Z\right)=C_{IoU}(\hat{X},Z)+\lambda C_{v}\left(\mathbb{Z},Z\right)$$

where $\hat{X}\in R^{N\times 7}$ is the set of object state estimations and $Z\in R^{M\times 5}$ is the set of observations on the new time step. λ is a weighting factor. ℤ contains the trajectory of observations of all existing tracks. $C_{IoU}\left(\cdot ,\cdot \right)$ calculates the negative pairwise IoU (intersection over union) and $C_{v}\left(\cdot ,\cdot \right)$ calculates the consistency between the directions of (i) linking two observations on an existing track and (ii) linking a track’s historical observation and a new observation.

#### 3.2.3. Trajectory Management

The management of trajectories involves three tasks, including trajectory creation, updating, and deletion. After completing pig data association matching, trajectories need to be updated for tracking pigs in the next frame. The management of trajectories includes the three following steps.

(1) For target pigs that fail to match, a new tracker is created for them, a new ID is assigned, and the information from the current detected pig is utilized for prediction in the next frame. For successfully matched tracking trajectories, we assign the detected pig to the ID of the successfully matched tracking target and update its trajectory information. The formula for updating the trajectory is as follows in Equations (5)–(7).

(5) $$K_{t}=P_{t|t-1}H_{t}^{T}{\left(H_{t}P_{t|t-1}H_{t}^{T}+R_{t}\right)}^{-1}$$

(6) $$x_{t|t}=x_{t|t-1}+K\left(z_{t}-H_{t}x_{t|t-1}\right)$$

(7) $$P_{t|t}=\left(I-K_{t}H_{t}\right)P_{t|t-1}$$

where K is the output values of the KF operation, R is the observation noise, H is the observation model, z is the observation value, t represents the current step, and t−1 represents the previous time step. Equation (5) is used to calculate the Kalman gain to estimate the significance of errors, while Equations (6) and (7) are used to compute the mean and covariance values for updating trajectories.

(2) If a trajectory can be associated again after a period of being untracked, we employ the ORU module to reduce the accumulated error during the tracking loss process. First, a virtual trajectory is constructed for the object, starting from the last detection before tracking loss and ending at the newly matched detection. By using the last-seen observation before being untracked as $z^{t1}$ and the observation triggering the re-association as $z_{t2}$, the virtual trajectory ($\left({\bar{z}}_{t}\right)$) is denoted as in Equation (8).

(8) $${\bar{z}}_{t}=Traj_{virtual}\left(z_{t1},z_{t2},t\right),t1<t<t2$$

Then, along the trajectory of ${\bar{z}}_{t}\left(t1<t<t2\right)$, we run the loop of predicting and re-updating. The re-update operation is as in Equation (9).

(9) $$re-update\left\{\begin{array}{l} K_{t}=P_{t|t-1}H_{t}^{T}{\left(H_{t}P_{t|t-1}H_{t}^{T}+R_{t}\right)}^{-1}\\ {\hat{x}}_{t|t}={\hat{x}}_{t|t-1}+K_{t}\left({\bar{z}}_{t}-H_{t}{\hat{x}}_{t|t-1}\right)\\ P_{t|t}=\left(I-K_{t}H_{t}\right)P_{t|t-1} \end{array}\right.$$

(3) For tracking trajectories that fail to match, we temporarily retain the trajectory without updating its status and associate this trajectory with the detected targets in the next frame. If the trajectory exceeds the predefined frame count (set as a maximum of 30 frames in this paper) without successfully matching any detection targets, we delete this trajectory.

**Figure 5:**
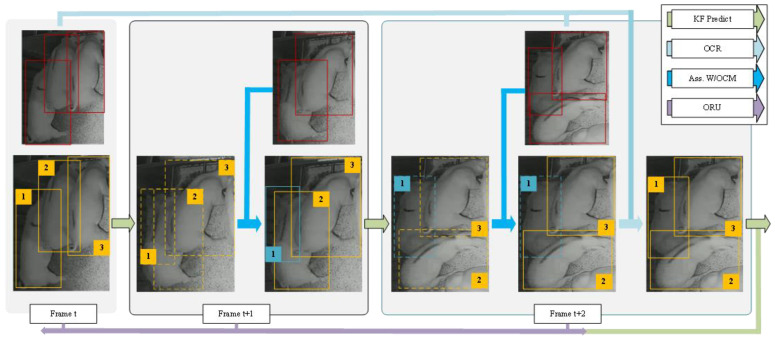
OC-SORT tracking process for pigs.

When a target is lost for re-tracking, OC-SORT can employ the OCR, OCM, and ORU strategy to recover the lost trajectory through virtual trajectories. Figure 5 illustrates the process of pig re-tracking using OC-SORT. The red boxes are detections, yellow boxes are active tracks, blue boxes are untracked tracks, and dashed boxes are the estimates from KF. During association, OCM is used to add the velocity consistency cost. Target #1 is lost on the frame t + 1 because of occlusions. But, on the next frame, it is recovered by referring to its observation of the frame t by OCR. It being re-tracked triggers ORU from t to t + 2 for the parameters of its KF. Through the OCR, OCM, and ORU modules in OC-SORT, the robustness during pig occlusion and nonlinear motion has been enhanced.

### 3.3. Pig Behavior Analysis Algorithm

We create and implement the pig’s quadruple behavioral time calculation algorithm (shown in Algorithm 1) based on the video sequence tracking results. The specific implementation steps in this algorithm are as follows:

(1) A behavioral statistics array named [$A_{1},A_{2},A_{3},A_{4}$] for each trajectory is constructed to save the four behavioral classifications information. And $A_{1},A_{2},A_{3}$, and $A_{4}$, respectively, represent the cumulative frame counts for the four categories of lying, standing, eating, and other behaviors.

(2) Based on the categories (stand, lie, eat, and other behaviors) for each pig ID, we create a frame counting array named $\left[a_{1},a_{2},a_{3},a_{4}\right]$. If the current detection box is identified as a “lie” behavior, the “lie” parameter ($a_{1}$) is set to 1, and the others are set to 0, and so on.

(3) After we associate detection boxes with tracking trajectories, we revise the values of the array $\left[A_{1},A_{2},A_{3},A_{4}\right]$ if the detection box and trajectory match successfully. The formula for the operation is as follows in Equation (10):

(10) $$\left[\begin{array}{l} A_{1new}\\ A_{2new}\\ A_{3new}\\ A_{4new} \end{array}\right]=\left[\begin{array}{l} A_{1}\\ A_{2}\\ A_{3}\\ A_{4} \end{array}\right]+\left[\begin{array}{l} a_{1}\\ a_{2}\\ a_{3}\\ a_{4} \end{array}\right]$$

The detection box is initialized as a new trajectory if it does not match the trajectory and its confidence score exceeds the threshold (equal to 0.7) [30], and the statistical array $\left[a_{1},a_{2},a_{3},a_{4}\right]$ parameters are set to 0. Finally, by accumulating the behavioral frame counts of all trajectories and dividing by the frame rate, we can obtain the duration of each pig behavior.
**Algorithm 1:** Pseudo-code of pig behavior analysis.Input: A video sequence V; object detector Det; tracking score threshold η is set 0.75; Frames per second Fps; Output: Tracks T of the video1Initialization: T←∅2for frame $f_{k}$ in V do3 $D\leftarrow Det\left(f_{k}\right)$4 Initialize time-count array including four elements for time statistics $a\leftarrow \left[0,0,0,0\right]$5 Set variable $category\_index\leftarrow D\left\{category\right\}$6 $a\left[category\_index\right]\leftarrow 1$7 Associate T with D using OC-SORT:8  if succeed to match then9   Call the update or re-activate function to update the status of tracks10   Set variable $A\leftarrow T\left[category\_time\_array\right]+a$11   $T\left[category\_time\_array\right]\leftarrow A$12  end13  if D failed to match and D>η then14   Call the function to create a new track.15   Initialize time-count array $A\leftarrow \left[0,0,0,0\right]$16   $T\left[category\_time\_array\right]\leftarrow A$17  end18End19$T\left[category\_time\_array\right]=T\left[category\_time\_array\right]/Fps$Return T


### 3.4. Evaluation Metrics for MOT

We select HOTA, MOTA, and IDF1 as evaluation metrics for pig MOT. The calculation of HOTA is shown in Equation (11), where DetA denotes the detection accuracy score, and AssA represents the association accuracy score. c is a point belonging to TP, from which a unique ground truth trajectory can be determined. $A\left(c\right)$ represents the association accuracy. TP refers to the number of true positive samples, FN refers to positive samples incorrectly predicted as negative, and FP refers to negative samples incorrectly predicted as positive.

(11) $$HOTA=\sqrt{DetA\cdot AssA}=\sqrt{\frac{\underset{c\in TP}{\Sigma }A\left(c\right)}{TP+FN+FP}}$$

The MOTA calculation is shown as Equation (12), where FP represents the total number of false detections in frame *t*. FN denotes the total number of missed detections in frame *t*, IDS refers to the number of ID switches that occur during tracking in frame *t*, and $g_{t}$ indicates the number of targets observed at frame *t*.

(12) $$MOTA=1-\frac{\sum_{t}\left(FP+FN+IDS\right)}{\sum_{t}g_{t}}$$

The IDF1 calculation is shown as Equation (13), where IDTP represents the total number of targets correctly tracked with an unchanged ID. IDFP represents the total number of targets incorrectly tracked with an unchanged ID. IDFN represents the total number of targets lost in tracking with an unchanged ID.

(13) $$IDF1=\frac{2IDTP}{2IDTP+IDFP+IDFN}$$

Additionally, the model performance is evaluated with the number of identity switches (IDS). Higher values of HOTA, MOTA, and IDF1, and a lower value of IDs indicate a better model performance.

## 4. Results and Analysis

### 4.1. Results Comparison of V8-Sort and Other MOT Methods

The comparative experimental results on the public and private datasets are shown in Table 3 using V8-Sort and three widely used object tracking algorithms, namely Trackformer, JDE, and TransTrack. In the public dataset, V8-Sort achieved a HOTA of 82.0%, a MOTA of 96.3%, and 22 IDs. Compared with the other three methods, the proposed method has the highest accuracy and the least number of IDs. The HOTA value of V8-Sort is 11.2%, 19.4%, and 18.2% higher than those of Trackformer, JDE, and TransTrack, respectively; MOTA is higher by 7.8%, 12.9%, and 17%, respectively; and there are 261, 451, and 501 fewer IDs, respectively. Moreover, in the private dataset, V8-Sort has a HOTA of 74.8%, a MOTA of 96.7%, and 17 IDs. Comparing Trackformer and TransTrack, the HOTA of V8-Sort is 1.3 and 17.3 percentage points higher, and the IDs are fewer by 24 and 378, respectively.

The results show that the tracking performance of V8-Sort outperforms those of Trackformer, JDE, and TransTrack. This is attributed to the fact that the observation-centered OC-SORT tracker introduces the momentum of object movement into the correlation phase and develops a pipeline that is less noisy and more robust to occlusions and no-linear motions as a means of enhancing the robustness and accuracy of tracking. Therefore, the V8-Sort method outperforms the other three algorithms, which are able to effectively handle issues such as target occlusion, intersection, and scale variations in complex scenes, thereby providing high-quality tracking results for pig behavior.

**Figure 6:**
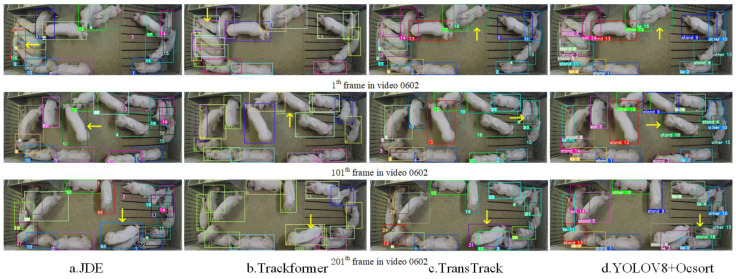
Comparison between V8-Sort and other tracking methods on public datasets.

Figure 6 illustrates the comparison of V8-Sort with other MOT methods on public datasets. In Figure 6, the yellow arrows indicate the maximum ID for each frame. It can be observed that JDE (a), Trackformer (b), and TransTrack (c) have the highest tracking IDs values of 93, 419, and 31 in frame 201 of video 0602. These numbers of IDs differ from the actual number of pigs in the pigsty (16), leading to frequent pig ID errors. In contrast, V8-Sort (d) consistently maintained the maximum number of IDs of 16 without any erroneous ID changes.

**Figure 7:**
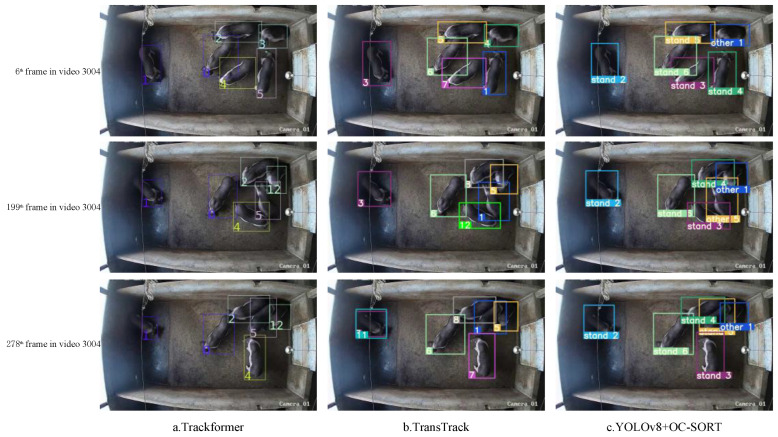
Comparison between V8-Sort and other tracking methods on private datasets.

Figure 7 illustrates the result of the comparison of V8-Sort with other MOT methods on private datasets. As depicted in Figure 7, Trackformer reaches a maximum tracking ID of 12 in frame 278 of video 3004, while TransTrack exhibits frequent erroneous IDs with a maximum number of tracked IDs of 12 and 11 in frames 199 and 278 of video 3004, respectively. V8-Sort maintains a stable maximum number of IDs of six. This stability is achieved because OC-SORT incorporates the motion trends of the targets into the similarity matrix, which helps to maintain the targets’ IDs across different frames and thus avoids ID error switches during the long-time tracking process.

### 4.2. Tracking Results of V8-Sort on One-Minute Videos Dataset

The results of the V8-Sort are shown in Table 4 and Table 5 on one-minute videos from the public and private datasets. It can be observed that, in the public dataset, the average MOTA, HOTA, and number of IDs for V8-Sort are 96.3%, 82%, and 22, respectively. In the private dataset, the average MOTA, HOTA, and number of IDs for V8-Sort are 93.7%, 74.8%, and 17, respectively. Among them, the highest MOTA is achieved in video 0802 and 3005, reaching 99.9%, while the lowest MOTA is in video 3009, at 71.2%. The main reason for the difference in MOTA among the different videos is due to the complex environmental conditions, such as video background, daytime or night time, sparse or dense scenarios, and the activity status of the pigs. In the daytime video 3009, the pigs are more active, resulting in a lower MOTA. In the night time video 0802, pig activity status is low, and the background is simple, leading to the highest MOTA. And, V8-Sort detects and tracks each pig in both daytime and night time scenarios.

**Figure 8:**
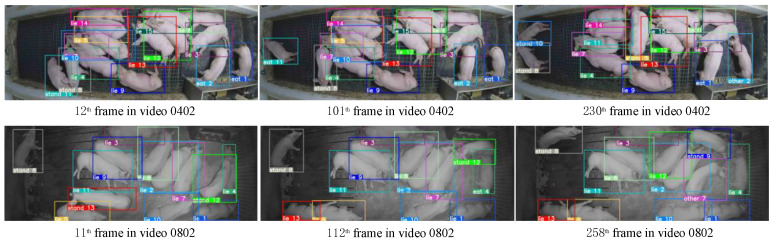
The visual results of V8-Sort on the public dataset.

**Figure 9:**
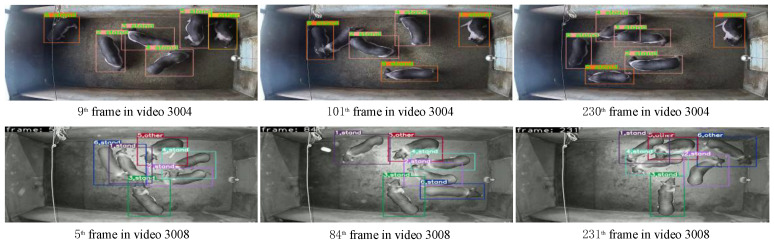
The tracking results visualization of V8-Sort on the private dataset.

To validate the feasibility and reliability of V8-Sort, we selected several segments from validation videos that were not used for training. From each of these segments, we chose three frames for presentation, and the tracking results are shown in Figure 8 and Figure 9, which, respectively, depict the visualization of tracking results for V8-Sort on public and private datasets. In the video segments 0402, 0802, 3004, and 3008, the maximum numbers of IDs of pigs in each frame are maintained at 15, 13, 6, and 6, respectively, which match the actual numbers of pigs. In conclusion, the aforementioned tracking results comprehensively demonstrate the outstanding accuracy and stability of the method proposed in this study within the complex environment of pig activity areas. By visualizing the tracking results, we can intuitively observe changes in pig behavior and trajectory evolution, enabling a deeper understanding of pig behavioral patterns and activity habits. This can provide valuable technological support for precision farming management of pigs.

### 4.3. The Long-Term Tracking Results of V8-Sort

The long-term tracking results based on V8-Sort are shown in Table 6. It can be observed that the average HOTA, number of IDs, MOTA, and IDF1 for videos 2001, 2002, 2003, 2004 and 3010 are 62.6%, 44, 93.1%, and 75.1%, respectively. There are significant differences between the tracking results of long-term and short-term videos. In the four 10 min videos, we achieve the highest values of HOTA, number of IDs, MOTA, and IDF1 (video 2002#) with 67.3%, 19, 97.1%, and 85.1%, respectively. In the 60 min video of 3010#, V8-Sort performs good, with HOTA of 69.0%, nine IDs, MOTA of 99.7%, and IDF1 of 75.1%. These disparities are primarily attributed to the duration and environmental conditions of the videos. Compared to 10 min videos, V8-Sort also makes the 60 min video achieve a better detection performance with a MOTA of 99.7%, and track the results with HOTA of 69%. The key reason is that, if a target is lost during long-term tracking, OC-SORT can employ the OCR, OCM, and ORU strategy to recover the lost trajectory through virtual trajectories and complete the re-tracking task. Therefore, V8-Sort achieves the more stable tracking of pig behaviors in long-term videos.

**Figure 10:**
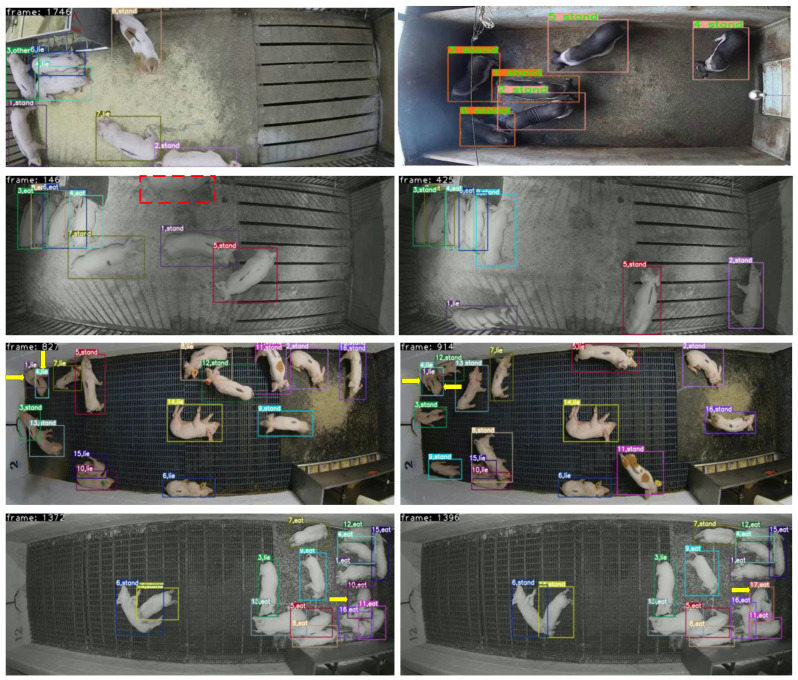
The visualization of long-term tracking results. (The first row shows the tracking results for videos 2001 and 3010, and the second, third, and fourth rows, respectively, depict the tracking results of two frames from videos 2002, 2003 and 2004).

The visual results of long-term tracking in different scenarios are shown in Figure 10. In videos 2001 and 3010, the pigs are sparsely distributed with minimal occlusion, and each pig’s behavior is accurately recognized. And, the maximum number of IDs consistently remains at eight and six, respectively, which both correspond to the actual number of pigs, reflecting stable tracking. However, the night time environment in video 2002 has led to occurrences of pig omissions, as indicated by the dashed boxes in the figure. Compared to videos 2001 and 2002, videos 2003 and 2004 exhibit a significant rise in the number of pigs. It can be observed that there are ID switches in frames 827 and 914 of video 2003, as indicated by the yellow arrows in the figure. In video 2004, substantial occlusion is present, resulting in a considerable number of omissions and ID switching issues. In these complex scenarios, the performance of the tracking system faces challenges, necessitating further algorithm optimization to enhance stability and accuracy.

### 4.4. Results of Behavior Analysis

**Figure 11:**
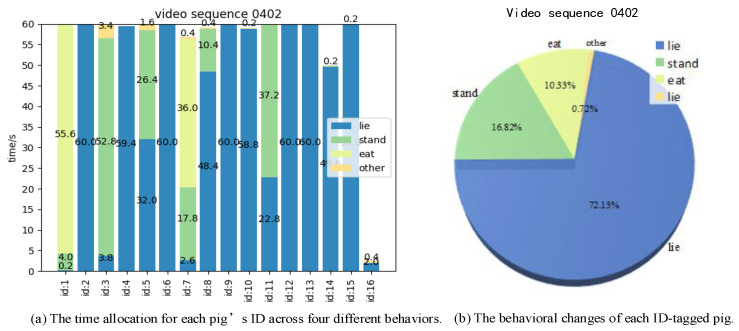
Time allocation and proportion of pig behaviors.

**Figure 12:**
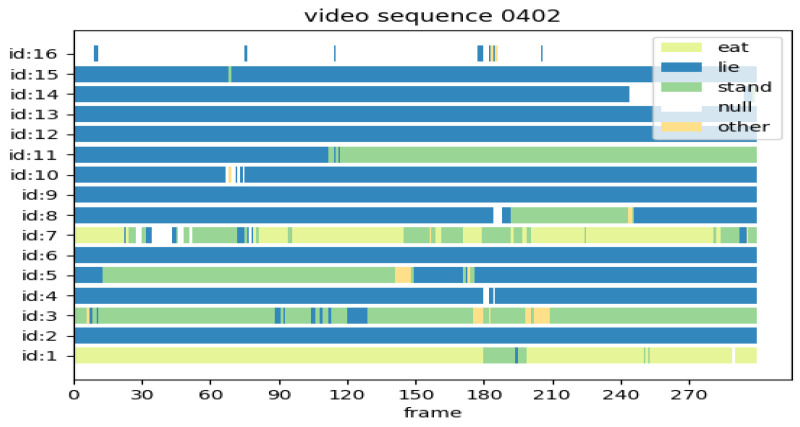
The proportional occurrence of the four behaviors.

Our proposed behavior analysis algorithm can record the duration of four types of behaviors for each pig and each pen based on behaviors classification and ID information to represent the pigs’ health status and welfare. For example, the analysis results of pig behaviors on test video 0402# are shown in Figure 11 and Figure 12.

In Figure 11a, the time allocation of each pig’s ID for four behaviors (lie, stand, eat, and other) is depicted. It can be observed that pigs with a different ID value exhibit variations in time allocation for each behavior, with “lie” and “stand” behaviors occupying a larger portion of the time, while “eat” and “other” behaviors are less prominent. Figure 11b illustrates the percentage of four behaviors of all pigs throughout the entire video segment, with different colors indicating each behavior. It is noticeable that the “lie”, “stand”, and “eat” behaviors occurred 72.13%, 16.81%, and 10.33% of the time for all pigs, and the ‘other’ behavior occurred the least at 0.72%. This indicates that the entire herd is in a healthy state. Figure 12 depicts the number of frames occupied by each pig ID, where the horizontal coordinate is the number of frames and the vertical coordinate is the pig’s ID value. It is worth noting that the behavior of some pigs varies frequently, such as those pigs with the IDs with 3, 5, 7, and there are also cases where several pigs engage in a single behavior for a long time. For example, pigs with the IDs 2, 6, 9, 12, 13, and 15 are consistently engaged in “lie” behavior.

Overall, the V8-Sort method can achieve the accurate recognition of pigs’ basic behaviors, playing a positive role in optimizing breeding environments and enhancing pig welfare, as well as contributing to the economic efficiency of the breeding industry.

## 5. Conclusions and Future Work

This paper proposes an algorithm for the identification and tracking of pig behaviors based on V8-Sort. The algorithm aims to decrease noisy interference and improve robustness to occlusions and nonlinear motions and explore the work of recovering the lost trajectory for long-term tracking. On the public dataset, V8-Sort achieved a HOTA of 82.0%, MOTA of 96.3%, and 22 IDs. Compared to Trackformer, JDE, and TransTrack, V8-Sort has shown improvements of 11.2, 19.4, and 18.2 percentage points in HOTA, and improvements of 7.8, 12.9, and 17 percentage points in MOTA, respectively. On the private dataset, V8-Sort has a HOTA of 74.8%, and 17 IDs. Comparing Trackformer and TransTrack, the HOTA is 1.3 and 17.3 percentage points higher, and the IDs are fewer by 24 and 378, respectively. In the long-term videos’ dataset, the average HOTA, number of IDs, MOTA, and IDF1 of V8-Sort are 62.6%, 44, 93.1%, and 75.1%, respectively. In conclusion, the V8-Sort can obtain higher accuracy and performance in pig behavior recognition and tracking tasks, which recover the lost trajectory during long-term tracking. This improves the accuracy and efficiency of the automatic monitoring of group-reared pigs under conditions of noisy interference and scenes with occlusions, providing a more intelligent and reliable solution for pig farming management.

However, the behaviors analyzed in the current study are relatively simple and basic, primarily focusing on obvious behavior patterns such as eating and resting. Important behaviors such as more complex social interactions, stress responses, and environmental adaptability have not yet been addressed. Through obtaining the group-housed behaviors analysis using the MOT method in this paper, we can consider in future research to complete the accurate discrimination of pig health status and welfare by analyzing herd behaviors, including daily and abnormal pig behaviors. In addition, by integrating advanced sensors, data analysis and machine learning techniques, it is possible to establish predictive models for correlating the health status of pigs with each other based on their behaviors. By monitoring key information such as the pigs’ activity levels, feeding patterns, and movement trajectories, combined with physiological parameters like body temperature and heart rate, we can obtain the real-time tracking of the health conditions of the pigs to facilitate production management in the modern livestock industry.

## Figures and Tables

**Table 1:** Description of the test dataset.

Dataset	Video Number	Day	Night	Sparse	Time	Activity Level	Number of Pigs
Public dataset	0102	√	—	√	1 min	H	7
	0402	√	—	×	1 min	M	15
	0502	—	√	√	1 min	M	8
	0602	√	—	×	1 min	H	16
	0702	√	—	×	1 min	M	12
	0802	—	√	×	1 min	L	13
	0902	√	—	×	1 min	M	14
	1002	—	√	×	1 min	M	14
	1102	√	—	×	1 min	H	16
	1202	√	—	×	1 min	L	15
	1502	—	√	×	1 min	M	16
	2001	√	—	√	10 min	L	7
	2002	—	√	√	10 min	H	8
	2003	√	—	×	10 min	L	16
	2004	—	√	×	10 min	M	15
Private dataset	3001	√	—	√	1 min	L	10
	3002	√	—	×	1 min	M	11
	3003	—	√	×	1 min	M	11
	3004	√	—	√	1 min	H	6
	3005	√	—	√	1 min	M	6
	3006	√	—	√	1 min	M	6
	3007	√	—	√	1 min	M	6
	3008	—	√	√	1 min	L	6
	3009	—	√	√	1 min	H	6
	3010	√	—	√	60 min	H	6

**Table 2:** Overview of the YOLOv8n layer functions.

Layer Name	Description
Stem layer	Initial layer for feature extraction and input processing.
Stage layer1	Processes the input with convolutional layers for feature refinement.
Stage layer2	Further refines features, capturing more complex patterns.
Stage layer3	Continues to extract and enhance feature representations.
Stage layer4	The final stage of the backbone, preparing features for the neck module.
TopDown layer1	Upsamples features for better spatial resolution.
TopDown layer2	Continues to upsample and merge features from different scales.
Down Sampl0	Reduces spatial dimensions for processing efficiency.
Bottom Up layer0	Integrates features from previous layers for enhanced information.
Down Sample1	Further downsampling to balance speed and accuracy.
Bottom Up layer1	Merges features, ensuring a comprehensive representation.
ConvModule	Applies convolution operations for feature extraction.
Con2d	Standard convolution layer for additional feature processing.
Bbox.Loss	Computes the loss related to bounding box predictions.
Cls.Loss	Computes the loss for classification accuracy.

**Table 3:** Comparison of V8-Sort with other MOT methods.

Video Sequence	Algorithm	HOTA/%↑	IDs↓	MOTA/%↑	IDF1/%↑	FP↓	FN↓
Public datasets	Trackformer	70.8	283	88.5	79.5	1048	3719
	JDE	62.6	473	83.4	71.2	3323	3455
	TransTrack	63.8	523	79.3	71.2	3627	4910
	**V8-Sort**	**82.0**	**22**	**96.3**	**96.8**	**658**	**953**
Private datasets	Trackformer	73.5	41	95.7	86.9	426	463
	TransTrack	57.5	395	82.1	67.2	1292	2279
	**V8-Sort**	**74.8**	**17**	**93.7**	**93.1**	**143**	**1232**

The “↑” indicates that a higher value is better, while “↓” indicates that a lower value is preferable. and bolded results represent the method used in this study.

**Table 4:** The tracking results of V8-Sort on the public dataset.

Video Squences	HOTA/%↑	IDs↓	MOTA/%↑	IDF1/%↑	FP↓	FN↓
0102	85.4	2	98.2	97.1	1	35
0402	84.3	0	95.4	97.7	137	68
0502	84.0	10	99.4	99.7	10	4
0602	73.8	0	90.6	91.9	275	165
0702	83.7	0	98.1	99.0	13	55
0802	91.9	0	99.9	99.9	0	3
0902	85.8	0	97.5	98.7	45	61
1002	72.2	0	92.2	96.0	93	236
1102	83.1	5	97.6	92.8	29	83
1202	81.6	5	95.0	95.6	26	193
1502	77.3	0	98.4	99.2	29	50
Total/average	82.0	22	96.8	96.8	658	953

The “↑” indicates that a higher value is better, while “↓” indicates that a lower value is preferable.

**Table 5:** The tracking results of V8-Sort on the private dataset.

Video Squences	HOTA/%↑	IDs↓	MOTA/%↑	IDF1/%↑	FP↓	FN↓
3001	78.0	2	97.0	97.1	11	87
3002	72.0	2	95.4	89.8	22	129
3003	70.1	3	93.0	93.0	27	202
3004	71.6	5	94.3	91.6	33	149
3005	82.0	0	99.9	99.9	0	2
3006	81.0	0	91.3	95.5	2	154
3007	82.6	0	97.6	98.8	18	25
3008	84.1	0	99.9	99.9	1	0
3009	52.3	5	71.2	70.6	29	484
Total/average	74.8	17	93.7	93.1	143	1232

The “↑” indicates that a higher value is better, while “↓” indicates that a lower value is preferable.

**Table 6:** The long-term tracking results based on the V8-Sort.

Video Squences	HOTA/%↑	IDs↓	MOTA/%↑	IDF1/%↑	FP↓	FN↓
2001	56.7	19	97.1	67.6	189	391
2002	67.3	29	90.4	85.1	291	1986
2003	61.2	73	93.6	73.0	1235	1741
2004	59.0	91	84.6	74.5	1737	5436
3010	69.0	9	99.7	75.1	75	201
Total/average	62.6	44	93.1	75.1	705	1951

The “↑” indicates that a higher value is better, while “↓” indicates that a lower value is preferable.

## Data Availability

All relevant data are included in the article.
